# Exploring mood disorders and treatment options using human stem cells

**DOI:** 10.1590/1678-4685-GMB-2023-0305

**Published:** 2024-07-01

**Authors:** Autumn Hudock, Zaira Paulina Leal, Amandeep Sharma, Arianna Mei, Renata Santos, Maria Carolina Marchetto

**Affiliations:** 1 University of California San Diego, Department of Anthropology, La Jolla, CA, USA.; 2 The Salk Institute for Biological Studies, Laboratory of Genetics, La Jolla, CA, USA.; 3 Université Paris Cité, Institute of Psychiatry and Neuroscience of Paris (IPNP), INSERM U1266, Signaling Mechanisms in Neurological Disorders, Paris, France.; 4 Institut des Sciences Biologiques, Centre National de la Recherche Scientifique (CNRS), Paris, France.

**Keywords:** Human disease modeling, major depressive disorder, treatment-resistant depression, bipolar disorder, induced pluripotent stem cells

## Abstract

Despite their global prevalence, the mechanisms for mood disorders like bipolar disorder and major depressive disorder remain largely misunderstood. Mood stabilizers and antidepressants, although useful and effective for some, do not have a high responsiveness rate across those with these conditions. One reason for low responsiveness to these drugs is patient heterogeneity, meaning there is diversity in patient characteristics relating to genetics, etiology, and environment affecting treatment. In the past two decades, novel induced pluripotent stem cell (iPSC) research and technology have enabled the use of human-derived brain cells as a new model to study human disease that can help account for patient variance. Human iPSC technology is an emerging tool to better understand the molecular mechanisms of these disorders as well as a platform to test novel treatments and existing pharmaceuticals. This literature review describes the use of iPSC technology to model bipolar and major depressive disorder, common medications used to treat these disorders, and novel patient-derived alternative treatment methods for non-responders stemming from past publications, as well as presenting new data derived from these models.

## Introduction

In 2006, Shinya Yamanaka and his graduate student Kazutoshi Takahashi discovered how to convert mouse fibroblasts into induced pluripotent stem cells (iPSCs) (Takahashi and Yamanaka, 2006). This study was shortly followed by the finding that human skin cells could also be reprogrammed into iPSCs (Takahashi *et al*., 2007). With time, researchers were able to coax differentiation from human induced pluripotent stem cells (iPSCs) into other cell types, such as neuronal progenitor cells (NPCs) and neurons, providing a tool to study neurological disease pathogenesis and mechanisms in targeted cell types for specific disorders, such as major depressive disorder (MDD) or bipolar disorder (BD) (Chen et al., 2014; Vadodaria et al., 2019a,b, 2021; Lu et al., 2023). Cells that were usually inaccessible, like neurons from patients with mood disorders, can now be generated using iPSC reprogramming technology derived from somatic tissues such as blood and skin (Otte et al., 2016; Vieta et al., 2018). The lack of understanding of the cellular and molecular pathology in mood disorders has contributed to the inefficiency of diagnostic tools and current treatment options available for the patients (Wong et al., 2010). 

**Figure 1 -:**
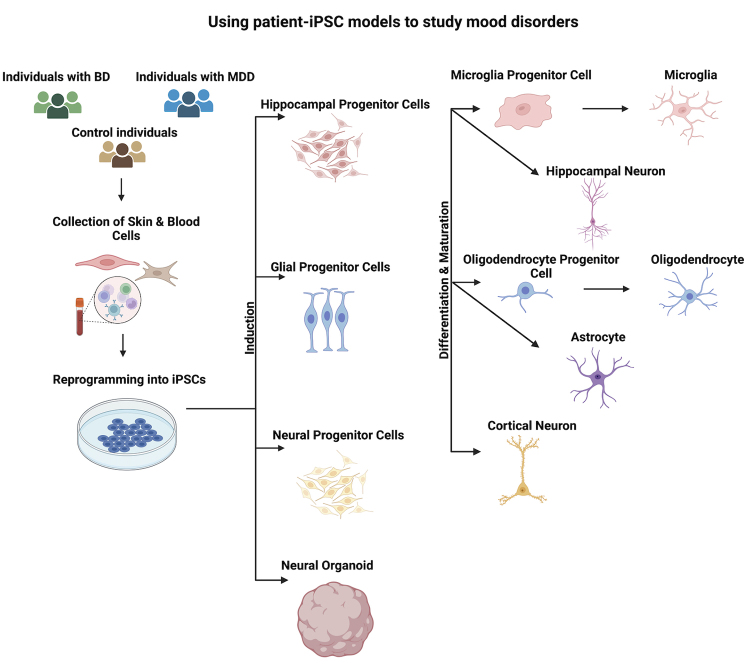
Using patient-iPSC models to study mood disorders. Patient skin or blood cells are collected and reprogrammed into iPSC using previously established protocols (see methods). The iPSCs are then differentiated into neuronal or glial progenitor cells in 2 or 3D cultures and matured into functional neurons and glial cells. These cells can then be used to study the disease pathophysiology, response to inflammatory stimuli and drug screening for novel therapeutic compounds. The graph represents the protocol of establishing cell lines in tissue culture settings to study mood disorders affecting the brain, beginning with the recruitment of cells, the process of reprogramming cells into iPSCs, and differentiating them into various neuronal cell types.

iPSC technology can help explore the molecular mechanisms and etiology of these disorders and test alternative treatment methods noninvasively (Vadodaria et al., 2016). Using patient-derived cells acts as a human disease model that can facilitate the exploration of alternative treatment options for those individuals who do not respond to or tolerate conventional pharmacological treatments available (Vadodaria *et al*., 2019a,b; Mishra et al., 2021). For mood disorders, drug responsiveness can be highly variable due to factors related to patient diversity and genomics. Exploring these differences using patient-derived iPSC models can help shed light on drug efficacy and understandings of cellular pathways related to pharmaceuticals used to treat mood disorders in an individualized capacity. This review explores the history of using iPSC technology to study BD and MDD cellular pathology and research that invokes the use of common medications for these disorders (Figure 1 and Tables 1 and 2). We present novel data showing the anti-inflammatory effects of apigenin on control and BD patient iPSC models. Lastly, we delineate the alternative medications and therapies used to treat these mood disorders and the challenges and limitations of modeling polygenic psychiatric conditions using patient-derived iPSCs.

**Table 1 -:** Induced pluripotent stem cells in the study of bipolar disorder: a chronological table of exploratory and treatment-based studies.

Study Type	Treatment	Cell/ Tissue Type(s)	iPSCs Relevance	Findings	Reference
Exploratory	N/a	iPSCs Neurons	Investigation of the cellular behavior and developmental pathways of iPSC derived neurons from BD patients and controls	-Increased membrane receptors and ion-controlled genes were noted in BD patient derived neurons compared to controls	Chen et al. (2014)
Exploratory	N/a	Postmortem cerebral tissue iPSCs Neurons NPCs	Examination of the expression of hsa-miR-34a in postmortem tissue and various IPSCs derived cell types	-Increased expression of microRNAs in BD patient derived iPSC cultures and postmortem cerebral tissue	Bavamian et al. (2015)
Exploratory	N/a	iPSCs NPCs Neurons	Models of iPSCs, NPCs and neurons of a 1^st^ degree family cohort analyzed using microarray technology to investigate the existence of global gene expression patterns in individuals with BD	-Differently expressed genes were observed during iPSC to NPC differentiation in BD patients -Enrichment of genes correlated with cell cycle regulation and homeostasis	Kim et al. (2015)
Exploratory	N/a	iPSCs Neurons NPCs	A family-based paradigm model was used to examine iPSCs lines from brothers with DB compared to non-BD parents	-NPCs and neuronal cells derived from BD patients showed phenotypical differences in channeled subunits and Wnt pathway components related to neuroplasticity	Madison et al. (2015)
Exploratory and Treatment	Lithium	iPSC-derived neurons	Study of phenotypical differences in neurons derived from iPSCs generated from lithium responder and non-responder patients and controls	- Discovered that BD derived neurons were hyperexcitable compared to control neurons -Lithium reversed hyperexcitability only on neurons derived from lithium responders	Mertens et al. (2015, 2016)
Treatment	Lithium	iPSC-derived neurons	Study of neurons derived from BD lithium responder patients to gain insight to BD molecular mechanisms and lithium targets	-Lithium acts on CRMP2 which alters neuronal cytoskeletal dynamics and spine formation in BD iPSC derived neurons	Tobe et al. (2017)
Exploratory	N/a	iPSCs NPCs	Examination of transcriptomes during the transition from iPSCs to NPCs in BD patients and controls using RNA sequencing	-Increased expression of genes related to inflammation in BD-derived NPCs -The most significantly differentially expressed genes were NLRP2 and genes associated with GABA and dopamine receptor canonical pathways	Vizlin-Hodzic et al. (2017)
Exploratory	N/a	iPSC-derived neurons	Modeled and compared activity of neurons derived iPSCs from lithium responders and non-responders BD patients	-Created a model to predict if a patient will respond to lithium or not with a 92% accuracy rate	Stern et al. (2018)
Exploratory	N/a	iPSC-derived neurons	Study of the behavior of neurons of hippocampus CA3 and dentate gyrus and motor neurons from lithium responders and non-responders BD patients	- Hyperexcitability is dependent on the type of neurons and unstable in neurons derived from lithium non-responders	Stern et al. (2020a,b)
Exploratory	N/a	Organoids	Exploration of functional mechanisms in BD by comparing BD-derived and control organoids	-BD derived patient neurons expressed upregulation of genes involved in neurogenesis, cell adhesion, synaptic morphology, and function and a downregulation of genes involved in immune signaling along with a reduction in mitochondria-associated endoplasmic reticulum membranes (MAMs) -BD derived organoids responded differently to electrical stimuli but similarly to non-BD derived models in the absence of electrical stimuli	Kathuria et al. (2020)
Treatment	Lithium	iPSC-derived NPCs and lymphoblastoid cell lines	Examination of cellular phenotypes of lithium responder and non-responder BD patients compared to healthy controls	-*In vitro* lithium treatment of BD derived iPSC lines encouraged cell proliferation, enhanced the expression of BCL2 and GSK3B and lowered mitochondrial membrane potential (MMP) - Low MMP was reversed using *in vitro* lithium exposure but remained unaffected in clinical lithium therapy treatment	Paul et al. (2020)
Treatment	Valproate	iPSCs	Examination of how valproate interacts with iPSCs from lithium responders and non-responders	-Valproate rescued dysregulation of cell proliferation and cell death in lithium responders and non-responders	Paul et al. (2020)
Exploratory	N/a	iPSC-derived astrocytes	Generated astrocyte models derived from iPSC from BD patients and controls to examine inflammation related phenotypes	-IL-6, a proinflammatory cytokine, was upregulated in iPSC-derived astrocytes from BD patients compared to controls -When BD iPSC derived astrocytes were co-cultured with neurons, there was a reduction in neuronal activity	Vadodaria et al. (2021)
Exploratory and Treatment	Lithiumand Valproic Acid	iPSC-derived neurons	Examined lithium resistance in iPSC-derived neurons derived from lithium non-responder patients with B	-NR neurons displayed less activity of the Wnt/β-catenin signaling pathway and a significant decrease in *LEF1* expression	Santos et al. (2021)
Exploratory	N/a	Stem Cells Neurons NPCs	Mapped the presence and arrangement of lithium ions in cells	-Unveiled the mechanisms of lithium ion distribution in multiple cell types including patient derived cells from individuals with BD to further explore and understand lithium- response pathways	McGhee et al. (2021)
Exploratory	N/a	iPSC-derived neurons	Examined the circadian-rhythm regulation in neurons derived from iPSCs of BD lithium responder and non-responder patients	- BD neurons showed differences in circadian-rhythm regulation with the most significant difference seen in lithium non-responders	Mishra et al. (2021)
Treatment	Trimetazidine	iPSC-derived neurons and astrocytes	Study aimed to repurpose trimetazidine to treat bipolar depression	- Trimetazidine increased the production of ATP, which can be deficient in BD patients, by altering cellular metabolic processes	Bortolasci et al. (2023)
Treatment	Lithium	iPSC-derived cortical spheroids	Examination of phenotypic effects of prolonged lithium exposure on corticoid spheroids derived from BD patients	-BD patient-derived corticoid spheroids exhibited transcriptional profiles with enriched differentially expressed genes associated with kidney function and ion homeostasis when exposed to chronic lithium treatment	Osete et al. (2023)
Treatment	Apigenin	iPSC-derived astrocytes	Tested the effects of apigenin, a neuroprotective and anti-inflammatory compound, on stimulated iPSC-derived astrocytes from BD and control individuals to examine alternative treatment methods for bipolar disorder	-BD and control astrocytes experienced an increase in the percentage of IL-6 expressing astrocytes irrespective of the proinflammatory stimuli used (IL-1b or TNF-a) - Both BD and control astrocytes showed higher sensitivity to proinflammatory cytokine IL-6 compared to TNF-a -Apigenin treatment successfully counteracted the inflammatory response in BD and control astrocytes	This study

Abbreviations: 3D: three-dimensional, ATP: adenosine triphosphate, BCL2: B-cell lymphoma 2, BD: bipolar disorder, CRMP2: collapsin response mediator protein-2, DEGs: differentially expressed genes, GABA: gamma-aminobutyric acid, GSK3: glycogen synthase kinase-3, hsa-miR: human microRNAs, IL-1b: interleukin-1-beta, IL-6: interleukin-6, iPSCs: induced pluripotent stem cells, LEF1: lymphoid enhancer-binding factor 1, Li: lithium, MAMs: mitochondria-associated endoplasmic reticulum membranes, MMP: mitochondrial membrane potential, NLRP2: NLR family pyrin domain containing 2, NPCs: neuronal progenitor cells, NR: non-responder, RNA: ribonucleic acid, TNF-a: tumor necrosis factor alpha, Wnt: wingless-related integration site

**Table 2 -:** Induced pluripotent stem cells in the study of major depressive disorder: a chronological table of exploratory and treatment based studies.

Study Type	Treatment	Cell/ Tissue Type(s)	Involvement of iPSCs in Study	Findings	Reference
Exploratory and Treatment	Sertraline	HPCs	**Exploratory**: To examine the effects of chronic stress, a known cofounder of depression, researchers exposed HPCs from healthy individuals to cortisol and dexamethasone (or dex, synthetic cortisol) **Treatment**: The HPCs were then treated with sertraline, the most prescribed SSRI for MDD	**Exploratory**: -Healthy HPCs exposed to dex and cortisol exhibited a decrease in cell proliferation and neuronal differentiation -Increased expression of genes involved in cell cycle inhibition **Treatment**: -Increase in glucocorticoid receptor transcription rate -Alterations in neurogenesis associated gene expression profiles	Anacker et al. (2011)
Exploratory	N/a	HPCs	This study explored the effects of proinflammatory cytokine interleukin-1b (IL-1b) on neuronal generation	-Increase in transcripts for kynurenine 3-monooxygenase (KMO) enzyme during cell differentiation -Blocking KMO resulted in the restoration reduced neurogenesis caused by IL-1b exposure	Zunszain et al. (2012)
Exploratory	N/a	HPCs	Examined the effects of cortisol exposure in MDD vs non-MDD hippocampal progenitor cells	-Increase in cell proliferation -Decrease in neurogenesis	Anacker et al. (2013a)
Treatment	Glucocorticoid regulated kinase 1 (SKG1)	HPCs	Explored the effects of glucocorticoid regulated kinase 1 (SKG1) on HPCs following cortisol exposure	-The effects of cortisol exposure in HPCs can be mitigated by SKG1	Anacker et al. (2013b)
Treatment	Sertraline	hAD-SCs	Examined the changes in human adipose-derived stem cells after sertraline treatment	-Increase in proliferation	Razavi et al. (2014)
Treatment	Venlafaxine (SNRI) Eicosapentaenoic acid (EPA) Sertraline (SSRI) docosahexaenoic acid (DHA)	HPCs	HPCs were treated with IL-1b to induce inflammation followed by exposure of various forms of treatment for MDD to examine the difference in molecular mechanisms by treatment	-Venlafaxine and eicosapentaenoic acid had anti-inflammatory effects HPCs exposed to IL-1b -Sertraline and docosahexaenoic acid had pro-inflammatory effects on IL-1b treated HPCs -All treatments were associated with a decrease in NF-kB pathway activity	Horowitz et al. (2015)
Treatment	Paroxetine	Neurons	Explored the effects of paroxetine neuronal differentiation and cell proliferation on adipose-derived stem cells	-Paroxetine enhanced neurogenic differentiation and proliferation rate	Jahromi et al. (2016)
Treatment	IL-1b	HPCs	Treated HPCs with IL-1b to examine effects on the kynurenic pathway	-IL-1b exposure lead to a restoration of neurogenesis	Borsini et al. (2017)
Exploratory	N/a	HPCs	The effects of interferon-a to investigate the mechanisms of inflammation-induced depression	-Reduction in neurogenesis -Increased cell death -Increase in expression of ubiquitin-specific peptidase 18 (USP18) and interferon-stimulated gene 15 (ISG15) was responsible for the reduction of neurogenesis -Increased expression in interleukin-6 (IL-6) is responsible for the increase in cell death	Borsini et al. (2018)
Treatment	Ketamine	Neurons	Examined the effects of ketamine on prefrontal hippocampal neurons derived from MDD patients	-Ketamine increased structural plasticity in MDD derived dopaminergic neurons	Cavalleri et al. (2018)
Exploratory	Ketamine	Neurons	Examined current literature on ketamine exposure on iPSCs to treat MDD	-Glutamate burst induced structural plasticity	Collo and Merlo Pitch (2018)
Exploratory	N/a	HPCs	Exposure of serum derived from patients with MDD on HPCs from non-MDD study participants	-Treated HPCs showed an increase in cell apoptosis and a decrease in neurogenesis matching that of MDD patient derived HPCs -Increase in apoptosis and decrease in neurogenesis exacerbated following interferon-a treatment	Borsini et al. (2019)
Treatment	Ropinirole and Pramipexole	Neurons	Examined the effect of ropinirole and pramipexole on neurons derived from patients with treatment resistant depression	-Ropinirole and pramipexole regulated structural plasticity in neurons derived from treatment resistant depression patients	Collo et al. (2018b)
Treatment	Serotonin and Latuda	Neurons	**Treatment (Serotonin)**: Exposed neurons derived from non-SSRI responders to Serotonin to examine changes in activity **Treatment (Latuda)**: Exposed SSRI non-responders to Latuda, an antipsychotic drug) to examine changes in activity	-Serotonin: Increase in activity related to the upregulation of excitatory serotonergic receptors (5-HT7 and 5-HT2A), -Latuda rescued hyperactivity in SSRI non responders	Vadodaria et al. (2019a)
Exploratory and Treatment		iPSC-derived serotonergic neurons	**Exploratory**: iPSCs from SSRI responders, non-responders and individuals with no history of MDD were juxtaposed to study differences in morphology, action and behavior **Treatment**: Treated cells with SSRIs to study the differences in the expression of serotonergic genes between the controlled and patient groups	-**Exploratory**: No difference found in the expression of serotonergic genes -**Treatment**: In SSRI non-responders, the genes protocadherin alpha 6 and alpha 8 exhibited were differentially expressed compared to SSRI responders	Vadodaria et al. (2019b)
Exploratory	N/a	HPCs	Examined the effects of IL-6 on HPCs from MDD and non-MDD derived HPCs	-Pro and anti-inflammatory effects on HPCs based on levels and presence of other cytokines	Borsini et al. (2020)
Treatment	Paroxetine	Oligodendrocytes	Examined the risk of neurotoxicity caused by SSRI medication; iPSC derived oligodendrocytes were exposed to MDD treatment paroxetine	-Reduction in neurite outgrowth -Cell population decrease	Zhong et al. (2020)
Treatment	Bupropion	Cortical neurons	Examined the effects of bupropion on cortical neurons derived from MDD bupropion responders compared to a controlled group	-Cortical neurons derived from MDD patients showed a difference in synaptic connection, gene expression and morphology	Avior et al. (2021)
Exploratory	N/a	Glial cells	Explored the effects of chronic cortisol exposure on glial cells from MDD patients	-Differentially expressed genes related to ion homeostasis, G-protein-coupled receptors (GPCR), and synaptic signaling	Heard et al. (2021)
Exploratory	N/a	Neurons	Examined neurons derived from MDD patients and compared them to a control	-MDD patient derived neurons demonstrated functional and bioenergetic differences -Increased electrical activity -Change in sodium ion channels	Triebelhorn et al. (2022)
Exploratory	N/a	Forebrain organoids	Examined the difference in morphology, behavior, and function of corticoid organoids derived from MDD patients with a history of attempted suicide	-GABAergic interneurons and ventral forebrain organoids form MDD patients exhibited an increase in neuronal firing -Difference in neuronal morphology -Decrease in calcium signals -The dysregulation in neuronal morphology and behavior may be due to the decreased expression of 5-HT2C, a serotonergic receptor	Lu et al. (2023)

Abbreviations: 5-HT2A: serotonergic receptor 2A, 5-HT2C: serotonergic receptor 2C, 5-HT7: serotonergic receptor 7, GABA: gamma-aminobutyric acid, GPCR: G-protein-coupled receptors, hAD-SCs: human adipose-derived stem cells, HPCs: hippocampal progenitor cells, IL-1b interleukin-1-beta, IL-6: interleukin-6, ISG15: interferon-stimulated gene 15, KMO: kynurenine 3-monooxygenase, MDD: major depressive disorder, NF-kB: nuclear factor kappa b, SKG1: serum and glucocorticoid-regulated kinase 1, SNRI: serotonin and norepinephrine reuptake inhibitor, SSRI: selective serotonin reuptake inhibitor, USP18: ubiquitin-specific peptidase 18

## Bipolar Disorder (BD)

BD is a psychiatric diagnosis given to individuals who experience sustained symptoms such as mood swings and periods of manic, hypomanic, and major depressive episodes (American Psychiatric Association, 2013). It is often characterized as a mood disorder and is split into two main types: type I and type II. Type I is correlated to higher occurrences of manic episodes, and type II emphasizes a higher likelihood of depressive episodes. Diagnostically, there are other subtypes of BD, such as substance/medication-induced BD and cyclothymic BD (American Psychiatric Association, 2013). The disorder affects millions, about 2% of the global population (Haggarty et al., 2021). Bipolar disorder is thought to impact neuronal proper maturation early during an individual’s development, even though the reported average age of onset is around 20 years of age (Vieta et al., 2018). 

The necessity to address viable diagnostic and treatment alternatives for bipolar disorder is urgent, as BD can lead to further health complications that surpass mood swings and depressive episodes (Leboyer et al., 2012). People with BD have higher chances of developing cardiovascular disease in youth and adulthood (Goldstein et al., 2015), as well as chronic inflammation leading to diabetes and hypertension (Goldstein *et al*., 2009). BD can also impair cognition, including memory, reaction time, and executive function (Cullen et al., 2016). Antipsychotic medication has also been linked to cognitive impairment for those with BD (Cullen *et al*., 2016). These impairments lead to disability, which can be detrimental for the individual - affecting financial, familial, and societal aspects of one’s life (Gilman, 2002). For those with BD, survey data points to a gap of about five years between the arrival of symptoms and when they receive a diagnosis and treatment options (Dagani et al., 2017) using traditional mental health care infrastructure.

Bipolar disorder is correlated to both genetic and environmental risk factors, such as family history of the disorder and childhood trauma (Vieta et al., 2018). BD is also the most heritable psychiatric disorder at around 85% prevalence (Vieta *et al*., 2018). Studies looking at families with a history of BD have found that genetic risk factors are associated with the disorder’s development (Haggarty et al., 2021). These genetic risk factors are not explicit biomarkers of bipolar disorder but provide insight into the disease’s etiology and pathogenesis, pointing to an early role in neuronal development. The manifestation of BD in the brain seems to rely on dysregulation of genes involved with cell signaling pathways, calcium signaling, inflammatory responses, histone and immune pathways, and microRNA and hormone pathways (O’Dushlaine et al., 2011; Forstner et al., 2015). In the following section, case studies of iPSCs models of BD will be discussed and contextualized in the greater framework of modeling mood disorders using a human-centered approach and how these studies can address the gap of understanding for BD treatment, pathogenesis, and neurobiology.

## Modeling BD using iPSCs

The paper by Chen et al. (2014) is one of the first studies using iPSCs to model BD. They investigated the developmental pathways and cellular behavior of patient-derived iPSCs from a group diagnosed with BD in comparison to healthy individuals and found that the patient-derived neurons expressed more membrane receptors and ion control genes compared to control neurons. Dysregulation of these genes has consequences for central nervous system (CNS) function and calcium signaling (Brini et al., 2014), which only further supports the necessity of establishing models to investigate the developmental pathways affected by the disorder. A following study (Mertens et al., 2015) supported the previous results (Chen *et al*., 2014), finding that there was increased ion channel expression for those with BD and that NPCs and neuronal cells of affected people displayed changes in Wnt (wingless-related integration site) and GSK3 (glycogen synthase kinase-3) signaling pathways, compared to controls. 

Subsequent iPSC studies demonstrated BD’s early role in development. Another study (Kim et al., 2015) used a cohort of patients who are known to be genetically isolated, belonging to the Old Order Amish group. The researchers established models of iPSCs, NPCs, and neurons of the first-degree family member cohort by comparing models for those in the population with type I bipolar disorder and those without it. To meaningfully compare the two groups, the team utilized microarray analyses to gain insight into BD biology in the brain. The goal of this study was to ultimately investigate global gene expression patterns for those affected by BD. The results showed that in the iPSC to NPC stage, there were differentially expressed genes (DEGs) with enhanced enrichment of genes that are correlated to cell cycle regulation and homeostasis. 

Another iPSC study revealed a hyperactive phenotype induced by increased evoked action potentials and increased calcium transients for BD patient-derived neurons (Mertens et al., 2015). Modeling BD using iPSCs has revealed identifiers that can predict lithium responsiveness for patients. For example, Stern et al. (2018), when modeling BD using iPSCs for lithium responders and non-responder cohorts, found that these two different neuron populations were so different that they could be investigated using solely electrophysiological properties, and generated a model that can predict a new patient’s possibility for lithium responsiveness with an accuracy of about 92%. Subsequent studies modeling BD (Stern *et al*., 2020a,b) affirmed the observed hyperexcitability phenotype of BD dentate gyrus hippocampal neurons, and the unique hyperexcitability found specifically in CA3 pyramidal neurons from lithium-responders and not found in lithium non-responders, as well as provided further evidence that BD works along potassium currents and sodium channels. 

MicroRNAs (miRNAs) are small, non-coding RNAs of 18-23 nucleotides that post-transcriptionally regulate gene expression (Bartel 2009). miRNAs are highly expressed in the brain and have recently emerged as essential regulators of neuronal development, differentiation, and plasticity (Miller and Wahlestedt, 2010; Xu et al., 2010; O’Connor et al., 2012; Alural et al., 2017). Bavamian et al. (2015) identified increased expression of human (hsa)-miR-34a in postmortem cerebellar tissue from BD patients, as well as in BD patient-derived iPSC-neuronal cultures. Hsa-miR-34a targets multiple genes implicated as genetic risk factors for BD, including ankyrin-3 (ANK3) and voltage-dependent L-type calcium channel subunit beta-3 (CACNB3). These data uncover the role of hsa-miR-34a in regulating multiple genes in BD and highlight the importance of miRNAs as potential targets for the development of novel BD therapeutics.

Brain organoids are a three dimensional culture of patient-derived iPSCs that recapitulate early stages of neuronal development, both functionally and structurally. Kathuria et al. (2020) focused on the functional aspects of BD using organoids from patient-derived iPSCs. The team generated organoids from eight patients with BD (type I), as well as organoids for eight control individuals. Their investigation using gene set enrichment analysis shows that genes involved in cell adhesion, neurogenesis, and synaptic morphology and function are upregulated in BD patient-derived neurons, and genes involved in immune signaling are downregulated for those same patients. Gene ontology (GO) showed that mitochondria-associated endoplasmic reticulum membranes (MAMs) are structurally different and reduced in the organoids of BD patients when compared to controls. This provides evidence for the idea that endoplasmic reticulum (ER) and mitochondria interactions are dysregulated in BD patients, affecting basic cellular processes. Additionally, the study (Kathuria *et al*., 2020) featured microelectrode arrays of nine-month-old organoids of healthy and BD patients, demonstrating that the BD patient models had functional differences in how they responded to electrical stimuli but had similarities when they were at a baseline without stimulation. 

Several lines of evidence suggest a link between imbalanced inflammatory signaling and BD (Munkholm et al., 2013; Najjar et al., 2013). BD patients show a higher prevalence of comorbid diseases with an inflammatory component, such as cardiovascular disease, diabetes, and immune-related ‘metabolic syndrome’ (Cassidy et al., 1999; Ösby et al., 2001; Weiner et al., 2011; Leboyer et al., 2012). Vadodaria et al. (2021) compared entire transcriptomes of a cohort of healthy and BD patients, revealing that a pro-inflammatory cytokine known as interleukin-6 (IL-6) was upregulated in BD patient-iPSC-derived astrocytes compared to controls. The subsequent response of BD astrocytes to another pro-inflammatory cytokine, interleukin-1b (IL-1b), revealed a unique transcriptional response to inflammation, with further increased secretion of IL-6 that directly and negatively impacted the activity of co-cultured neurons. Another study (Vizlin-Hodzic et al., 2017) detected increased expression of inflammation-related genes in BD patient-derived NPCs. The group detected the most highly significant differentially expressed gene as NLR family pyrin domain containing 2 (NLRP2), followed by DEGs associated with dopamine and gamma-aminobutyric acid (GABA) receptor canonical pathways. These studies support the hypothesis that dysregulated expression of genes involved in the inflammatory system occurs during early fetal brain development of BD patients and can contribute to impaired neuronal function. These findings collectively highlight the promising potential of investigating anti-inflammatory compounds as complementary therapeutic approaches for BD.

**Figure 2 -:**
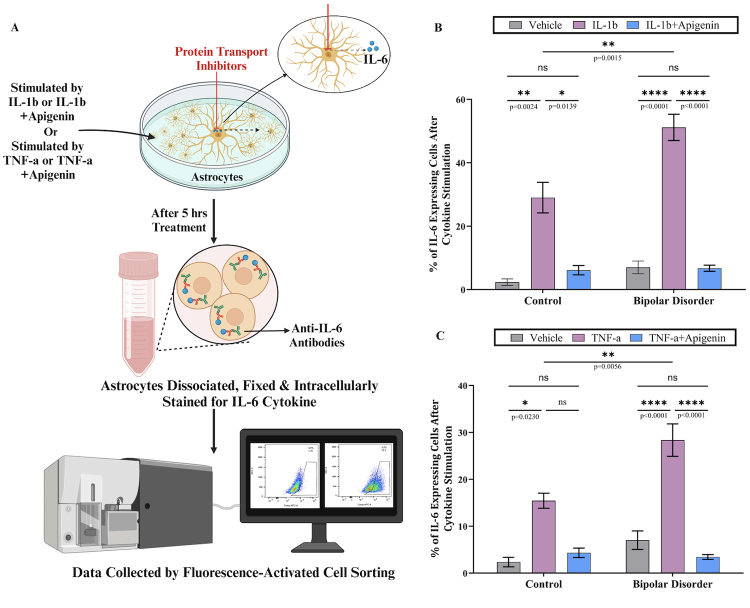
Apigenin attenuated the inflammation response more effectively in iPSCs-derived astrocytes from BD patients. (A) Schematics of investigating anti-inflammatory attributes of a flavonoid (Apigenin). iPSC, glial progenitor cells (GPCs) and astrocytes derived from three neurotypical and six BD donors, described previously (Santos et al., 2017; Vadodaria et al., 2021) were used in this study. This assay was previously described by Vadodaria *et al*. (2021). Briefly, pro-inflammatory stimuli were added to 4-week-old astrocytes for 5 hours, either by using recombinant human IL-1b (10 ng/mL) or recombinant human TNF-a (50 ng/mL); PBS was used as a non-stimulated control. To mitigate the inflammatory response, astrocytes were simultaneously treated with 20 μM Apigenin. BD GolgiPlug and BD GolgiStop were added to the treatments to inhibit extracellular protein secretion. Flow cytometry was used to quantify IL-6 producing cells. Astrocytes were dissociated using a 1:1 ratio of accutase/papain followed by staining with Zombie UV fixable Viability kit. The BD Cytofix/Cytoperm and BD Perm/Wash kits were used for fixing and permeabilizing the cells. Subsequently, IL-6 cytokine was labeled with APC conjugated anti-IL-6 antibody in BD Perm/Wash for 20 min. Data collection and analysis were conducted using a BD CantoII cytometer and FlowJo software, respectively. Negative gating controls for anti-IL-6 were done in non-stimulated samples stained with rat IgG1-APC antibody. The IL-6 positive cells were quantified by normalizing the data with non-stimulated cells from the vehicle treated cells. Data are from two biological experiments with technical replicates (3). (B & C) Bar graph representing the quantification of astrocytes expressing IL-6 cytokine post 5 hours of exposure to Vehicle or IL1-b or IL1-b+Apigenin (B) or Vehicle or TNF-a or TNF-a+Apigenin (C), mean ± SEM. Two-way Anova test was used to determine the statistical significance.

Flavonoids, bioactive compounds found in various plant-based foods with remarkable antioxidant properties, gained substantial attention, positioning them as promising candidates for managing inflammatory disorders (Dourado et al., 2020). We tested the effects of one such compound, apigenin, a widely distributed bioflavonoid known for its neuroprotective (Nabavi et al., 2018) and anti-inflammatory properties (Li et al., 2016), on the stimulated astrocytes derived from iPSCs from both control subjects and individuals diagnosed with BD. Astrocytes generated from BD patients and healthy subjects were treated with pro-inflammatory cytokines (IL-1b or TNF-a) as described previously **(** Santos et al., 2017; Vadodaria et al., 2021 **)** in the presence or absence of apigenin (Figure 2A).

Our results show that pro-inflammatory stimuli with either IL-1b or TNF-a increased the percentage of astrocytes expressing IL-6 in both the control and BD groups that was reversed by apigenin treatment (Figure 2B and 2C). As observed before the pro-inflammatory response of BD astrocytes was significantly higher than the controls (Figure 2B and 2C). This data provides compelling evidence for exploring anti-inflammatory compounds as a complementary therapeutic approach to addressing BD.

## Using iPSCs to understand lithium treatment for BD 

Lithium (Li), a commonly prescribed mood stabilizer, has been used to treat bipolar disorder for over 70 years, but the mechanisms behind how it reduces manic episodes for some BD patients were elusive to researchers prior to iPSC modeling (Malhi et al., 2013). Studying the Li response in BD iPSC-derived neurons allowed researchers to correlate *in vitro* data with clinical metrics (Paul et al., 2020). iPSC models of BD demonstrated that lithium works in some patients by rescuing dysregulation of key processes on the cellular level (Stern et al., 2020a,b). Initial *in vitro* studies by Mertens et al. (2015) and Stern *et al*. (2018) showed that BD-derived iPSCs were hyperactive compared to controls, and only a subset of the lines responded to Li treatment. Importantly, the subset of BD neuronal lines that responded to lithium treatment were derived from BD patients who were also responsive to lithium treatment in the clinical setting, confirming that patient drug responsiveness could be recapitulated in an *in vitro* disease modeling setting using patient-derived cells. Mertens *et al*. (2015) cultured iPSC-derived neurons from individuals with BD and revealed a hyperactive action-potential phenotype displaying increased evoked action potentials by electrophysiology and increased calcium transients. The hyperexcitability state was attenuated by lithium treatment but only for neurons derived from individuals who previously responded to lithium. 

A following iPSC study (Tobe et al., 2017) found that in the BD brain, there is sometimes a response to lithium that alters collapsin response mediator protein-2 (CRMP2) phosphorylation, and in turn that affects the cytoskeleton organization responsible for the modulation of neuronal networks. A newly published paper featuring cortical spheroids (a 3D neuronal cell culture) noted that chronically (exposed for a duration of one month) lithium-treated cortical spheroids from patients with BD showed a transcription profile with enrichment in differentially expressed genes involved in processes such as sodium ion homeostasis and kidney function (Osete et al., 2023). This paper offers more insight into how lithium treatment duration and application in the rescue of gene expression profiles of BD patients is processed at the cellular and genetic level, investigating both diagnosis and treatment.

Using iPSC models, Mishra et al. (2021) demonstrated that neurons derived from BD patients had differences in circadian rhythm compared to healthy controls and that these differences were the most stark in the cohort of lithium non-responders. In the same year, McGhee et al. (2021) established a sensor that can visualize and capture the presence and arrangement of lithium ions in cells, which can be performed on various cell types, including stem cells, neurons, and NPCs. The sensor can also be applied to control cell lines, not just patient-derived cells from those with BD. The distribution of lithium ions was previously unknown and presented another barrier for understanding lithium-response pathways. 

Since lithium is effective in some patients with BD but not others, researchers continue to parse out the differences in these neuronal models to locate particular regions of the genome associated with lithium responsiveness (Stern et al., 2018; Mishra et al., 2021). Paul et al. (2020) established cell cultures of NPCs and LCLs (lymphoblastoid cell lines) for healthy controls, responders to lithium, and non-responders to lithium and found that cell proliferation is much higher for those with BD. However, lithium treatment did not rescue this phenomenon. BD patients also had increased cell death compared to healthy controls. When treated with lithium, only lithium responders were rescued. In addition, cells with high mitochondrial membrane potential (MMP) were less present in BD NPCs, than the control NPCs, revealing that cell viability is an essential aspect in lithium response. Modeling lithium-response pathways using iPSCs has proven useful in piecing together the mechanisms involved in BD and the treatment of the disorder. IPSC models of BD also highlight the demographic of those with the disorder who do not respond to some prescription drugs used to treat the disorder, such as lithium. 

## Exploring alternative treatments for BD using iPSCs

Although lithium helps reduce the occurrence of manic episodes, it has side effects that could lead to long-term gastrointestinal damage (Osete et al., 2021), and the medication favors individuals with acute bipolar disorder, a less severe manifestation of BD. Additionally, once an individual receives treatment for their diagnosis, they may not experience a reduction in symptoms, since drug effectiveness works differently depending on genetics and neurobiology (Santos et al., 2021). Therefore, there is a pressing need to explore alternative drugs and their effects on people with BD that stretch beyond lithium. 

Using iPSCs has allowed researchers to test drug effectiveness in a manner that does not lead to potential harm or burden for the patient and by providing *in vitro* insight for the exploration of other medications in the treatment of BD. For example, one study looked at mitochondrial respiration and the effects of three mood stabilizers (lithium, valproate, and lamotrigine) on iPSC models of lithium-responders, lithium-non-responders, and a non-treated cohort (Osete et al., 2021). All three drugs induced a transcriptional signature primarily enhanced in ribosomal and oxidative phosphorylation pathways. The researchers noted that lithium-treated responder NPCs had a better oxygen consumption rate. When exposed to valproate, the non-treated cohort NPCs demonstrated maximum respiration and reserve capacity, resulting in a better oxygen consumption rate. 

Another study using lithium alternatives (Paul et al., 2020) found that valproate rescues cell death and dysregulation of cell proliferation in both lithium non-responder and responder iPSC models. Santos and colleagues (Santos et al., 2021) established iPSC models of non-responders to lithium and found that lymphoid enhancer-binding factor 1 (*LEF1*) gene was less expressed in BD patient neurons. They also found that the Wnt/B-catenin signaling pathway was different for non-responders comparatively. For controls, the team found that when the expression for *LEF1* gene decreased, there was an increase in the hyperexcitability of neurons, which affirms the correlation between the two. They also found that valproic acid (a form of valproate) was able to rescue hyperexcitability in non-responders. This study provides evidence that *LEF1* gene is a possible target for drug therapy due to the causative relationship between valproic acid and *LEF1* expression demonstrated in the article. 

A study by Bortolasci et al. (2023) published a report using iPSC-derived neurons and astrocytes to screen for target genes associated with BD by producing a gene expression signature after exposure to BD medication and found that trimetazidine was an appropriate match in their analysis. The medication is known to work on the metabolic processes of the body and has a tendency to increase the production of adenosine triphosphate (ATP) (Şekeroğlu et al., 2021), and ATP is thought to be a component of BD defects (Jones et al., 2021). Published studies report that BD patients have abnormalities in mitochondrial respiration (Osete et al., 2021), a pathway that is known to be affected by trimetazidine. Although Bortolasci and colleagues (Bortolasci *et al*., 2023) did not strictly recapitulate the medication exposure in the iPSC models, the team was able to show that exposure to trimetazidine reduced BD-like symptoms in rats, showing the promise of iPSC modeling to address gaps in BD treatment plans. 

The necessity and practicality of exploring alternative treatments for those with BD has been highlighted by research invoking the use of iPSC models. These models have demonstrated valuable insight into the signaling pathways involved with mood stabilizer responsiveness, as well as differences present in cellular processes like mitochondrial respiration for lithium responders, and non-responders. 

## Major Depressive Disorder (MDD)

Individual costs and burdens of MDD are plentiful - however, maybe most pressing is that MDD is a leading cause of suicide in several countries (Vos et al., 2015). The traditional diagnostic process of MDD begins with an individual being referred to a specialized and licensed professional based on having shifts in mood and experiencing a depressive episode for longer than 14 days (American Psychiatric Association, 2013). These episodes are incredibly likely to return throughout one’s life but can be mitigated with pharmacological, therapeutic, and other combined and sustained medical intervention (Otte et al., 2016). 

Global studies show that women are twice as likely to have MDD onset after puberty, as men (Seedat et al., 2009). Similar studies have found that the average age of onset for MDD is around 25 years of age (Bromet et al., 2011). Cross-national studies point to specific risk factors for individuals who have developed MDD, including having experienced recent traumatic and adverse life events (Bromet *et al*., 2011). People who have experienced childhood trauma are twice as likely to develop MDD (Otte et al., 2016). Chronic stress is also a significant risk factor in developing major depressive disorder (Zunszain et al., 2012; Anacker et al., 2013b). Understanding the interactions of neurobiology, genetics, and environment for those with MDD is crucial to achieving more effective and accessible treatment options.

The current consensus of how MDD develops in an individual and the causes of the disorder are a culmination of genetic, psychological, and environmental factors (Bromet et al., 2011). MDD acts on the brain and most notably affects the process of neurotransmitters and synaptic activity in the forebrain region (Park et al., 2021). Neurotransmitters, like serotonin, norepinephrine, and dopamine, are essential for regulating the central nervous system (CNS) (Goldstein et al., 2015). SSRIs (selective serotonin reuptake inhibitors) and SNRIs (serotonin and norepinephrine reuptake inhibitors) are popular antidepressants used to treat MDD that act on neurotransmitters and can alter synaptic activity (Vadodaria et al. 2019a,b). However, around half of those with MDD do not respond to antidepressants and are subject to further risk factors contributing to the disorder, encouraging researchers to investigate alternative treatments for affected individuals (Taliaz et al., 2021). 

A significant portion of those diagnosed with MDD are thought to be genetically predisposed to the disorder, as MDD is known to affect entire families in some cases (Klein et al., 2001). Genetic studies have shown that MDD has a significant overlap with genes associated with other psychiatric disorders (Park et al., 2021). The genetics of major depressive disorder reveal polygenic risk scores on an individual basis, but there is no official manner of treatment or diagnosis provided through one’s genetics (Fabbri et al., 2020). The following section details how patient-derived iPSC modeling of neurons can be a pathway to research drug responsiveness and potential treatments in the case of MDD and may provide insight into the neurophysiological differences between individuals with or without the disorder.

## Modeling MDD using iPSCs

An early focus of iPSC studies on depression was capturing the effects of risk factors associated with the disorder, such as inflammation and chronic stress, often using exposure to cortisol and dexamethasone as a means to model depression in hippocampal progenitor cells (HPCs) from healthy individuals. One study found that when comparing exposure to low and high doses of cortisol on HPCs, there was an increase in cell proliferation of 16% and a decrease in neurogenesis but not an increase in astrocyte differentiation (Anacker et al., 2013a). Glucocorticoid hormones are known to be increased in rat models simulating stress and depression-like scenarios (David et al., 2009) and in blood samples from individuals with MDD (van Rossum et al., 2006), and too much of these hormones can halt neurogenesis (Snyder et al., 2011). Using HPCs, Anacker *et al*. (2013b) were able to identify that serum and glucocorticoid-regulated kinase 1 (SKG1) does have a role in the regulation of effects brought on by exposure to cortisol, revealing that inhibition of SKG1 may be a possible treatment pathway in the future for those with depression. 

A study by Heard et al. (2021) found that when chronically exposed to cortisol, glial cells (astrocytes) derived from patients with MDD have a host of differentially expressed genes compared to those with no history of the disorder. The differentially regulated genes in MDD were related to G-protein-coupled receptors (GPCR), ligand binding, synaptic signaling, and ion homeostasis. The data highlights astrocytes’ important role in the central nervous system in MDD under chronic stress conditions. The pro-inflammatory cytokine interleukin-1b (IL-1b) is induced in depressed patients, and depression is correlated with inflammation and reduced neurogenesis. Zunszain et al. (2012) aimed to recapitulate observations from rat models that demonstrated a reduced neuronal generation when exposed to IL-1b but using human hippocampal progenitor cells (HPCs). Investigating the response to IL-1b for the cell models, the researchers found that during cell differentiation, the exposure induced an increase in transcripts for the enzyme kynurenine 3-monooxygenase (KMO), mediated through the neurotoxic branch of the kynurenine pathway. They aimed to address this by blocking KMO and found that some aspects of the reduced neurogenesis affected by IL-1b were able to be rescued. 

The trend of investigating pathways involved in regulating inflammation-induced depression using iPSCs is also present in a string of experiments by Borsini and colleagues. One study used HPCs to model the effects of interferon-a in an *in vitro* setting (Borsini *et al*., 2018), noting that when interferon-a was administered to patients with viral hepatitis, there were positive effects on symptoms associated with depression. They found that after exposure to interferon-a, the hippocampal progenitor cells demonstrated a reduction in neurogenesis and increased cell death. In terms of molecular pathways activated, interferon-a exposure caused an increase in the expression of interferon-stimulated gene 15 (ISG15), ubiquitin-specific peptidase 18 (USP18), and interleukin-6 (IL-6), set off by signal transducer and activator of transcription 1 (STAT-1). ISG15 and USP18 were proposed to be responsible for the decrease in neurogenesis and IL-6 for the increase in cell death. Additional studies from Borsini and colleagues show that exposure of human hippocampal progenitor cells with serum from depressed subjects induces cell apoptosis and less neurogenesis than exposure to serum from non-depressed individuals, and these effects were exacerbated after treatment with interferon-a (Borsini *et al*., 2019). Subsequently, the authors also evaluate the bimodal action of IL-6 as having both pro- and anti-inflammatory actions on human hippocampal stem cell lines depending on its concentration levels and on the concomitant presence of other pro-inflammatory cytokines in the surroundings (Borsini *et al*., 2020). Together, these results provide evidence for the role of the individual’s systemic milieu in the regulation of hippocampal neurogenesis by inflammation and its influence on neuropsychiatric conditions.

In the last four years, studies have been published that offer more applicability to MDD, as they focus on using cell lines from individuals with depression as a point of comparison with healthy controls, and even further, responders and non-responders to SSRIs within the depressed cohorts. Vadodaria et al. (2019b) derived iPSCs and neurons from three individuals who responded extremely well to SSRIs, Escitalopram and Citalopram, three individuals who are highly treatment-resistant (non-responders to SSRIs Escitalopram and Citalopram), and three individuals who were established as controls with no history of depression. They compared the activity, behavior, and morphology of these groups to one another, as well as any changes reported once the cells were treated with SSRIs. Because the authors were interested in the neuronal processes involved in SSRI treatment, they used serotonergic neurons for the study, finding no differences in the expression of key serotonergic genes between patient and control groups. However, when looking at the entire transcriptome for these iPSC-derived neurons, the team found that the genes protocadherin alpha 6 (PCDHA6) and protocadherin alpha 8 (PCDHA8) were lowered in expression for SSRI non-responders, versus the control and responder groups. When the group tested knockout expression profiles of PCDHA6 and PCDHA8, they reported longer neurites correlated to serotonergic control neurons. This adds to the body of evidence suggesting that genes such as PCDHA6 and PCDHA8, and other protocadherin genes, may be responsible for the regulation of serotonergic neuron morphology (Katori et al., 2009), leading researchers to the conclusion that dysregulation of these genes is correlated to SSRI resistance in some manner (Vadodaria *et al*., 2019b). 

Another study by Vadodaria et al. (2019a) revealed that forebrain neurons derived from depressed patients with a history of no response to SSRIs show increased neuronal activity induced by the addition of serotonin (5-HT) to the culture media. The increased activity in non-responders was associated with the upregulation of excitatory serotonergic receptors (5-HT2A and 5-HT7). Blocking of the receptors using the atypical antipsychotic drug (*Latuda*) rescued the hyperactivity in SSRI non-responder neurons, showing a potential avenue for therapy in this group of patients (Vadodaria *et al*., 2019a). These studies highlight the importance of patient stratification based on pharmacological responsiveness to *in vitro* disease modeling as a tool for discovering disease-relevant mechanisms and neuronal phenotypes. 

Avior et al. (2021) demonstrated how bupropion affects patient-derived cortical neurons, specifically responders to bupropion, as a means to study individualized disease models of MDD. The study reveals differences in morphology, synaptic connectivity, and gene expression for those with MDD, leading to more robust evidence of biomarkers that can be used as drug response predictors in a personalized manner. Triebelhorn et al. (2022) showed that depressed patient neurons were different functionally and bioenergetically, including variance in sodium ion channels and increased electrical activity compared to controls. Lu et al. (2023) found that GABAergic interneurons and ventral forebrain organoids derived from MDD patients who have attempted suicide exhibit hyper neuronal firing, a decrease in calcium signal propagation, and differences in neuronal morphology compared to controls. They also found that the dysregulation in neuronal activity and morphology may be associated with decreased expression of serotonergic receptor 2C (5-HT2C) receptor. Using patient-derived cells affected with depression has allowed researchers to pursue a nuanced model of the manifestation and diversity of MDD in the human brain. 

## Using SSRIs, SNRIs, and iPSCs to understand MDD

A major part of investigating the pathways involved in MDD treatment relies on experiments documenting SSRI medication’s effects on human neuronal models. Anacker et al. (2011) implemented a range of tests on human HPCs treated with dexamethasone (or dex, synthetic cortisol), as well as cortisol, to mimic stress in an *in vitro* setting, being that chronic stress can induce depression. When the human HPCs were exposed to dex and cortisol, researchers found that there was a decrease in cell proliferation and neuronal differentiation, and genes involved in cell cycle inhibition were increased in expression. Adding the layer of treatment with the most commonly prescribed SSRI, sertraline, the HPCs mimicked the results of animal studies by demonstrating an increase in neuronal differentiation invoking a glucocorticoid-dependent pathway, an increase of 16% in immature doublecortin (Dcx)-positive neuroblasts, and an increase of 26% for mature microtubule-associated protein 2 (MAP2)-positive neurons. This paper outlines that sertraline can alter the gene expression profiles associated with neurogenesis and induce an increase in the glucocorticoid receptor (GR) transcription rate. In addition, exposure to sertraline also pointed to alterations of GR phosphorylation. 

A subsequent study using hAD-SCs (human adipose-derived stem cells) found that sertraline (SSRI) can promote cell proliferation but does not promote gliogenesis in these cultures (Razavi et al., 2014). Unlike previous studies (Anacker et al., 2011; Zunszain et al., 2012), Razavi *et al*. (2014) did not detect an effect on MAP2-positive neurons. A similar experiment noting the rescue effects that some antidepressants have regarding cell proliferation and differentiation tested a commonly prescribed antidepressant known as paroxetine on hAD-SCs (Jahromi et al., 2016). Their results revealed that paroxetine did alter the proliferation rate throughout cell culture, leading to neuronal differentiation, and the exposure induced an overall increase in Nestin and MAP2-positive neurons, as well as a reduction in glial acidic fibrillary protein (GFAP)-positive cells. 

A study by Horowitz et al. (2015) used IL-1b as an inflammatory stimulus and compared the response of the antidepressants and fatty acids on their ability to regulate the inflammation-immune response in HPCs. Venlafaxine (SNRI) and eicosapentaenoic acid (EPA) had anti-inflammatory effects. However, sertraline (SSRI) and docosahexaenoic acid (DHA) were both pro-inflammatory. While all treatments were associated with a decrease in NF-kB pathway activity, they were likely acting via different molecular mechanisms that resulted in either an anti- or pro-inflammatory downstream reaction. These findings caution that further characterization of the mechanism of actions of monoaminergic antidepressants and fatty acids is essential to understanding immune processes in depressed patients. Another study used HPCs and recorded exposure to IL-1b to test the potential rescue of dysregulation in the kynurenine pathway that leads to decreased neurogenesis linked to depression, using the antidepressants, sertraline, and venlafaxine, and the fatty acids, DHA, and EPA (Borsini et al., 2017). They rescued the reduction in neurogenesis, demonstrated by a decrease in MAP2-positive neurons and in Dcx-positive neuronal progenitors brought on by IL-1b exposure. To an extent, all compounds were able to promote a reduction of quinolinic acid levels brought on by IL-1b exposure, which further demonstrates a relationship with regulation involving the neurotoxic branch of the kynurenine pathway. 

A portion of the population on antidepressants includes people who can become pregnant. Individuals who regularly take antidepressants are not encouraged to stop them abruptly, even during pregnancy (Dubovicky et al., 2017). There is mixed evidence produced through rodent models on the possible effects of antidepressants on the fetal brain (Hutchison et al., 2021). However, the use of human iPSCs is an avenue of research that is well suited to investigate possible reactions of antidepressants during development, as a 3D model of neuronal cells known as a organoid can most efficiently mimic early stages of fetal development in humans (Di Lullo and Kriegstein, 2017). 

Interested in the developmental neurotoxicity produced by a common SSRI, using an established brain organoid derived from human iPSCs, Zhong et al. (2020) demonstrated that paroxetine (SSRI) affected a few aspects of neurotoxicity, including a population difference of oligodendrocytes in treated cells compared to controls, as well as a reduction in neurite outgrowth properties and expression of synaptic markers. Additionally, the cell population decreased between 40-75%, and the reductions of neurite outgrowth and synaptic marker expression were around 60% and 80%, respectively. This study outlines a reliable model that utilizes human cellular conditions to test antidepressant neurotoxicity during fetal development. Although using SSRIs and iPSCs to understand the mechanisms of MDD treatment has been insightful, the group of individuals presenting non-responsive to these traditional treatment methods also presents a pathway of drug discovery using novel human tissue culture technology. 

## Exploring alternative treatments for MDD using iPSCs 

Often, patients have to try multiple combinations of pharmaceuticals to find lasting relief from the symptoms of MDD (Otte et al., 2016). The side effects and low response rate tied to antidepressants highlight the need for alternative treatment options for those living with MDD (Taliaz et al., 2021). Treatment-resistant depression, TRD (a diagnosis referring to individuals with MDD who do not respond to other medications), has been correlated with chronic stress and dysregulation of structural plasticity in the brain for those affected (Collo et al., 2019b). However, the use of ketamine as a treatment option for those with treatment-resistant depression has found some success in addressing symptoms of MDD (Collo and Merlo Pich, 2018). This has encouraged researchers to use iPSC models to understand the drug’s effects compared to other compounds in the case of non-responders. 

Cavalleri et al. (2018) found that ketamine did increase structural plasticity in the dopaminergic (DA) neurons derived from mouse and human iPSCs. The consensus of the researchers, based on observations and previous findings, was that ketamine exposure was able to access pathways induced by a-amino-3-hydroxy-5-methyl-4-isoxazolepropionic acid receptor (AMPAR)-driven brain-derived neurotrophic factor (BDNF) and mammalian target of rapamycin (mTOR) signaling, in turn affecting structural neuroplasticity, in both mouse and human DA neurons. Another study found that ketamine may also affect extracellular signal-regulated kinase (ERK) pathways (Collo and Merlo Pich, 2018). Further research from the team demonstrated that glutamate synthase 1 (Glu1) and glutamate synthase 2 (Glu2) may have a role in regulating structural plasticity through dendritic upregulation, depending on ketamine exposure (Collo *et al*. 2018a, 2019a). Another study by Collo *et al.* (2019b) treated human DA neurons derived from iPSCs with ketamine and its active metabolite (2R,6R)-hydroxynorketamine ((2R,6R)-HNK) and found similar results to previous studies, demonstrating effects on regulating structural plasticity on more than the hippocampal and frontocortical circuitry of the brain, and providing evidence of alterations to the circuitry of the dopaminergic system. 

Cavalleri et al. (2018) found a correlation between ketamine effectiveness and fully intact D3 dopamine receptors, leading to antidepressant effects. The medications, ropinirole, and pramipexole, both typically prescribed to Parkinson patients, are thought to act on D2 and D3 receptors and may have a role in regulating structural plasticity (Varga et al., 2009), possibly offering relief for those with TRD, similar to the effects of ketamine exposure. Understanding the background of this research, Collo et al. (2018b) established two lines of iPSCs from donors that are healthy and unaffected by TRD and MDD, differentiating these cultures to midbrain dopaminergic neurons. Ultimately, they demonstrated that treating the human DA neurons with ropinirole and pramipexole assisted in regulating structural plasticity by invoking the use of D3 receptors and induction of the BDNF-TrkB and mTOR signaling pathways. Studies exploring the effects and mechanisms involved in MDD, and especially in treatment-resistant depression, have displayed an increase in understanding for the ways in which various compounds can be applied and tested for drug responsiveness for patients who have previously been labeled ‘non-responders’. 

## Limitations

Challenges presented in this field include access to mental healthcare, the ability to recruit diverse demographics for material collection, the scope of iPSC models, and the type of cell lines used in experiments. 2D iPSC models, such as monolayer and co-culture studies, lack situational context and interaction with other cell types that would occur naturally, such as interactions between glia and other brain tissue types that may be required to demonstrate neurogenesis efficiently (McNeill et al., 2020). Using 2D models derived from iPSCs tends to be more efficient and less time-consuming than establishing 3D models, such as organoids (Liu et al., 2022). However, 3D models can better replicate tissue interactions for *in vivo* studies but have proven to be difficult to standardize across the field of stem cell studies (Andrews and Kriegstein, 2022). Also, 3D organoid cultures might only partially capture the environment of the adult hippocampus, as these models best represent the early stages of neuronal development. Even the oldest brain organoids only resemble around three months of in-utero human fetal development (Osete et al., 2023). Due to this, these experiments lack the context of tissue age in correlation with disease onset in one’s life but represent appropriate models for fetal development.

Utilizing iPSCs as models for mood disorders share a familiar challenge along with animal models, in that it is difficult to gauge behavioral abnormalities when comparing data to humans living with mood disorders. For these reasons, readouts (parameters and/or rubric established for interpretation purposes) must be defined for iPSC models of mood disorders. Not having appropriate parameters for these readouts results in major challenges for exploring alternative therapeutic approaches for the treatment of mood disorders. These limitations remind us that iPSC models taken from patients with mood disorders are not the patients themselves, but rather, are a limited depiction of the disorder. 

Some studies lack an applicable diagnosis in their models, as they only utilize cells derived from healthy individuals rather than individuals who are diagnosed with a mood disorder they aim to model (Anacker et al. 2013a,b). In addition, studying cytokines can be tricky in cell culture settings, as cytokines are known to act differently on a tissue-by-tissue basis, and within different bodily regions, especially the brain (Munkholm et al., 2013). However, for decades, pharmaceutical companies have relied on using potentially flawed animal models from species that do not share remotely similar neurobiology with humans (Yin et al., 2016). So, regardless of these limitations, and with time and effort, the use of iPSC modeling represents a promise to help bridge the gap in diagnostic and treatment deficits for those with mood disorders and beyond.

## Discussion and Conclusion

**Figure 3 -:**
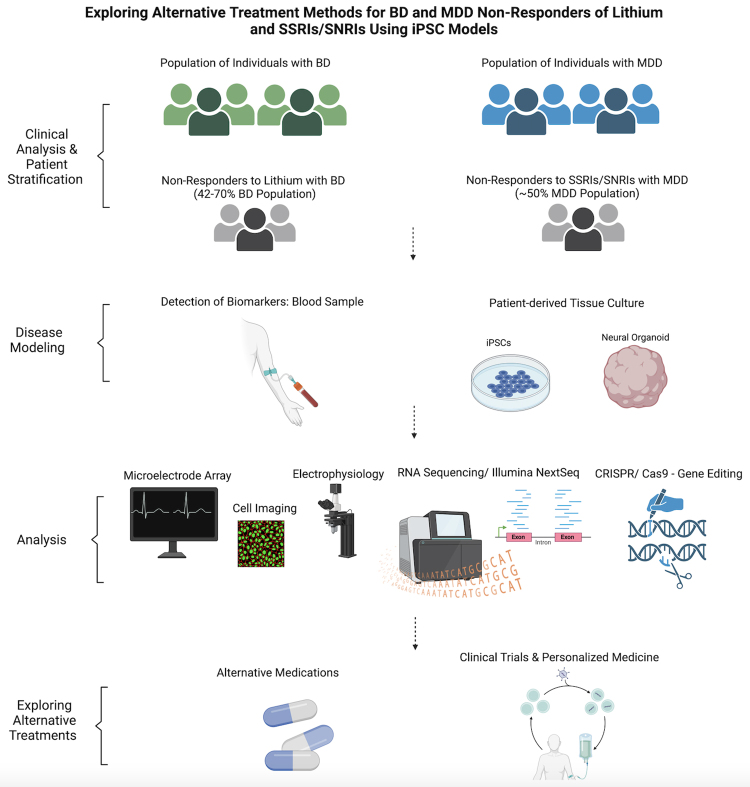
Exploring alternative treatment methods for BD and MDD non-responders to lithium and SSRIs/ SNRIs using iPSC models. The graph depicts the pathways that researchers have used to explore alternative treatment methods beyond lithium and SSRIs/SNRIs for non-responders with BD and MDD, as they make up about half of the population for those diagnosed with both disorders (Tighe et al., 2011; Taliaz et al., 2021). Beginning with the collection of cells, researchers can look for biomarkers associated with drug responsiveness, and model personalized neuronal models of patients that can be subjected to various forms of analysis. This data can contribute to the exploration of new compounds and alternative drugs already on the market, leading to relief for non-responders of traditional treatment methods.

The research tradition of BD and iPSC studies began with a focus on modeling the disorder directly by using cell lines from people with BD and comparing molecular and functional differences of neuronal cells with control groups (Chen et al., 2014; Bavamian et al., 2015; Kathuria et al., 2020). At the same time, researchers were interested in the genetic risk factors of BD, using patient-derived iPSC lines to compare across families with a history of the disorder (Kim et al., 2015; Madison et al., 2015). A subsection of this field is using iPSCs to model the pathways affected by medication in the treatment of BD, primarily lithium, being that it has a sustained history of prescribed use and partial success (Mertens et al., 2015; Tobe et al., 2017). The addition of analyzing target genes associated with BD and alternative treatment options using iPSCs creates a research model for drug effectiveness that does not subject participants to undue burden present in clinical trials (Figure 3) (Bavamian *et al*., 2015; Santos et al., 2021). Other studies using iPSC models of BD have demonstrated that inflammation plays a role in the manifestation and treatment of phenotypes associated with the disorder (Vizlin-Hodzic et al., 2017), along with various molecular and electrophysiological differences compared to control groups (Stern et al., 2018, 2020a,b; McGhee et al., 2021; Mishra et al., 2021; Vadodaria et al., 2021). More recently, researchers have used iPSC models to explore alternative pharmaceutical treatments for individuals with BD, focusing on lithium non-responders (Paul et al., 2020; Stern *et al*., 2020b; Osete et al., 2021; Bortolasci et al., 2023). In addition, the novel data in Figure 2 demonstrate that targeting inflammatory responses correlated to BD, using anti-inflammatory compounds such as apigenin, could provide an alternative treatment for people suffering from the disorder.

The initial studies attempting to model depression using iPSCs focused on analyzing antidepressant exposure on healthy cell lines to better understand the cellular and molecular mechanisms related to depression medication (Anacker et al., 2011; Zunszain et al., 2012; Razavi et al., 2014; Horowitz et al., 2015; Jahromi et al., 2016). The following research included a focus on modeling stress in healthy iPSCs to investigate the risk factors associated with developing depression (Anacker *et al*., 2013a,b). The efficacy of alternative medications for treatment-resistant depression is another area of study using iPSC models, and testing compounds such as ketamine and Parkinson medication (Collo et al., 2018a,b, 2019a,b). Some researchers have explored the use of omega 3s DHA and EPA as an anti-inflammatory therapeutic to address inflammation-induced depression (Horowitz *et al*., 2015; Borsini et al., 2017, 2018, 2020, 2021). 

The latest studies have established iPSC cell lines derived from people with an MDD diagnosis rather than healthy controls, which shows an emphasis on more reliable and practical evidence stemming directly from those with the disorder instead of an attempt to mimic depression-like symptoms using synthetic compounds (Avior et al., 2021; Heard et al., 2021; Triebelhorn et al., 2022; Lu et al., 2023). This research invokes the use and comparison of cells treated with SSRIs, SNRIs, and other compounds related to treating depression to investigate how non-responders, responders, and controls fare differently when exposed to different compounds (Horowitz et al., 2015; Vadodaria et al., 2019a,b). Animal models and post-mortem tissue analysis have been informative, but iPSC models offer an avenue for testing mechanisms and treatments for BD and MDD in a human and non-harmful context (Figure 3).
